# Use of Positive Predictive Value to Evaluate the Armed Forces Health Surveillance Division Brain Cancer Incidence Rules, Active Component Department of the Air Force Pediatric Dependent Population, January 1, 2010–December 31, 2020

**Published:** 2025-01-20

**Authors:** Sarah Fryman-Wynkoop, Crystal Tacke

**Affiliations:** 1 Epidemiology Consult Service, U.S. Air Force School of Aerospace Medicine, Wright-Patterson Air Force Base, OH

## Abstract

The Armed Forces Health Surveillance Division (AFHSD) uses a surveillance case definition to identify malignant brain tumors among U.S. active service members. This case definition was applied to the dependent pediatric population of the active component of the Department of the Air Force, which identified 179 malignant brain cancer cases. Those identified pediatric cases were reviewed using multiple data sources. The positive predictive value (PPV) of the AFHSD case definition was found to be 64.5% (95% confidence interval [CI], 55.9-72.5%). In 2019, Webber et al. reported a PPV of 84.3% for brain and other nervous system cancers among U.S. active component officers. The current pediatric study’s lower PPV suggests the case definition may be less effective for pediatric populations, indicating a need for refining surveillance methods for dependent populations. The AFHSD case definition was less effective at identifying malignant brain tumors in the active component Air Force pediatric dependent population, with a lower PPV compared to previous studies of the active component Air Force adult population. In addition, several cases were missed by the AFHSD rules.

**What are the new findings?:**

The PPV of the AFHSD case definition was lower when applied to the Air Force pediatric dependent population (64.5%; 95% CI, 55.9-72.5%) compared to the previously published PPV in the adult population (84.3%). There were an additional 16 cases of malignant brain tumors missed by initial screening utilizing AFHSD incidence rules.

**What is the impact on readiness and force health protection?:**

Chronic disease surveillance within the U.S. Department of Defense often relies on the Defense Medical Surveillance System for case identification. Accurate case definitions are essential to accurately identify cases. Further examination of the AFHSD malignant brain tumor case definition is recommended, to more effectively capture cases within the Department of Defense dependent population.

## 1 BACKGROUND

Standard surveillance case definitions and incidence rules by the Armed Forces Health Surveillance Division (AFHSD) are used for routine surveillance and descriptive epidemiological reporting of invasive cancers among active component service members (ACSMs). A malignant brain tumor case definition was developed in 2010 by AFHSD, the Office of the Assistant Secretary of Defense for Health Affairs, the U.S. Army Public Health Command, and the U.S. Military Cancer Institute.^1^ The case definition is based on ICD-9 and ICD-10 (International Classification of Diseases, 9th Revision and 10th Revision) codes, scientific literature, and previous AFHSD analysis. These case definitions can be applied to patient encounter data by utilizing diagnostic and treatment codes occurring within specified time periods to identify incident malignant brain tumor cases. Benign brain tumors are not included in this case definition and therefore should not be captured using this methodology.

In 2019, Webber et al. completed a review of multiple cancers in active component U.S. Navy, Air Force, and Marine Corps officers who entered service as company grade officers between January 1, 1986 and December 31, 2006.^2^ Of the 121 cases of brain cancer identified by the AFHSD incidence rules, 91 were confirmed brain cancer after chart review, yielding a positive predictive value (PPV) of 84.3%. Cases that were unable to be reviewed (n=13), due to absence of medical records, were not included in the calculation.^2^ Validating these case definitions in other populations, such as dependents and retirees, is important for epidemiological studies that use AFHSD case definitions to identify cases among all Military Health System (MHS) beneficiaries.

The current study was prompted by a review of pediatric brain cancer cases among an Air Force base population conducted by the U.S. Air Force School of Aerospace Medicine’s Epidemiology Consult Service.^3^ The review utilized the AFHSD surveillance case definition to identify cases of malignant brain tumors. The pediatric brain cancer case review included a detailed chart review, which discovered that a proportion of the cases identified by the AFHSD case definition were not malignant brain tumors, raising concerns about the PPV of the case definition. The objective of the current study is to determine the accuracy of the AFHSD case definition for identifying malignant brain tumors among the active component Department of Air Force (DAF) pediatric dependent population.

## 2 METHODS

The population of interest was the DAF pediatric dependent population, diagnosed with brain cancer between January 1, 2010 and December 31, 2020 (n=583,244). The cohort, defined as ages 0-19 years, follows the pediatric age grouping established by the Central Brain Tumor Registry of the United States.^4^ Potential brain cancer cases were identified utilizing the 3 case-finding methodologies outlined in the AFHSD case definition for malignant brain cancer: 1) hospitalization with malignant diagnosis in first position, 2) hospitalization with a ‘Z’ or ‘V’ code for anti-neoplastic treatment in first position and malignant diagnosis code in second position, or 3) ambulatory encounters (within 90 days) with malignant diagnosis in first or second encounters.^2^

The DMSS dataset includes both inpatient and outpatient encounters from military hospitals and clinics (i.e., direct care) as well as care provided at civilian facilities billed to TRICARE (i.e., purchased care). Each identified case was then reviewed by a team comprised of a physician and 3 epidemiologists who examined medical records from multiple systems including the Armed Forces Health Longitudinal Technology Application (AHLTA), Health Artifact and Image Management Solution (HAIMS), Military Health System GENESIS (MHS GENESIS), and Joint Legacy Viewer (JLV). This review extracted clinical notes, pathology reports, imaging results, and other relevant medical documentation to assess whether each individual had a clinician-confirmed diagnosis of primary malignant brain cancer.

Cases were stratified by type of care, either inpatient or outpatient, and direct or purchased, to determine where the largest portion of misclassification occurred. The PPV was calculated as the number of confirmed primary malignant brain cancer cases (i.e., true positives) divided by the sum of cases that were either primary malignant brain cancer cases (true positives) or confirmed as not primary malignant brain cancer cases (i.e., false positives). Cases that were unable to be reviewed, due to absence of medical records in AHLTA, JLV, or MHS GENESIS, were not included in the calculation. The Clopper-Pearson method was used to provide the 95% confidence interval (CI) based on the cumulative probabilities of the binomial distribution.

To identify potentially missed cases, all encounters were scanned for the presence of at least 1 malignant brain cancer ICD-9 or ICD-10 code in any of the first 10 diagnostic positions. Individuals flagged by this filter were further investigated through a detailed electronic health record (EHR) chart review. The same team (1 physician and 3 epidemiologists) completed this additional review, following the same chart review methodology.

## 3 RESULTS

The AFHSD case definition identified 179 potential cases of malignant brain tumors within the pediatric dependent population of the U.S. Air Force over an 11-year period of observation. Of those potential cases, 89 were confirmed as true positives, 49 as false positives, and 41 cases were not found or were unknown due to a lack of information in EHRs (excluded from PPV calculation). The overall PPV was calculated as 64.5% (95% CI, 55.9-72.4%) (**Table**).

Inpatient encounters had a higher true positive rate (66.4%) compared to outpatient encounters (21.2%). Within inpatient care, both direct care and purchased care had similar true positive rates (66.2% and 66.6%, respectively). Outpatient encounters showed a notably higher false positive rate (43.9%) compared to inpatient encounters (17.7%). Of the 46 cases identified from outpatient purchased care, 22 were classified as unknown due to insufficient information, 18 were determined to be false positives, and only 5 were confirmed as true positives.

The additional review, which scanned the remaining cohort (n=583,065 dependents) for at least 1 relevant ICD code within the first 10 diagnostic positions, identified 203 potential additional cases. Subsequent chart reviews of these cases confirmed 16 primary malignant brain tumors missed by the AFHSD rules. All 16 missed cases were from outpatient encounters. The reasons for these missed cases varied: 9 cases had a brain cancer ICD code in the first or second diagnostic position but did not have enough of these encounters; 3 cases had 3 outpatient encounters but were spaced 93 to 151 days apart; and 4 cases had the correct ICD codes but not in the first or second diagnostic position.

## 4 DISCUSSION

Several factors may explain the lower PPV observed in the current study compared to Webber et al. One notable difference is the higher percentage of potential cases in the current study that could not be reviewed due to missing data (22.3% compared to 14% in Webber et al.). This discrepancy is likely due to unique limitations associated with pediatric populations, which are more likely to be referred to specialized oncology centers, where claims may be processed through alternate systems, potentially bypassing standard DOD medical claims datasets. These referrals often result in cancer-related claim processing outside standard DOD medical claims datasets, contributing to the higher percentage of ‘unknowns’ in this analysis. Additionally, the ‘unknowns’ in this study were predominantly identified from outpatient encounters, which are subject to a high false positive rate. While Webber et al. acknowledged that the inclusion of these ‘unknowns’ could either increase or decrease PPV estimates, the current analysis suggests their inclusion likely contributed to the lower PPV observed in this study. These findings underscore the need to address gaps in data capture and variability in coding practices to improve the accuracy of surveillance case definitions, particularly for pediatric cancer cases.

The challenges in data capture and variability are further compounded by differences in the incidence and presentation of brain tumors in pediatric populations. Brain tumors are less common in children, with an incidence of 5.7 per 100,000 persons in children compared to 29.9 per 100,000 persons in adults.^5^ While brain tumors are the most frequent solid cancers observed in children, they often present with non-specific symptoms that may mimic more common childhood illnesses, increasing the likelihood of misdiagnosis or delayed diagnosis.^6,7^

An important limitation of the AFHSD surveillance definitions is reliance on ICD coding practices. Those definitions assume that cancer-related codes will appear in the first or second diagnostic fields, which may not always correlate with actual coding variability. This study demonstrates the limitation of that assumption, particularly in pediatric populations, where variability in provider coding practices and referral patterns may result in cancer diagnosis coding in less prominent diagnostic positions.

The additional descriptive analyses of this study examined whether chart review alone could have effectively identified primary malignant brain tumors. While chart review was essential for confirming diagnoses, it was the combination of systematic scanning of ICD-9 and ICD-10 codes within the first 10 diagnostic positions with chart reviews that enabled the identification of 16 additional cases of primary malignant brain tumors. This dual approach proved to be both efficient and feasible, especially compared to the impracticality of reviewing hundreds of thousands of charts manually (without utilizing any surveillance case definition).

All 16 missed cases were identified through outpatient encounters, however, which reveals specific limitations of the AFHSD surveillance case definition. Nine of those missed cases had a brain cancer ICD code in the first or second diagnostic position but failed to meet the required encounter frequency; 3 cases met the encounter frequency criterion but were too far apart (93 to 151 days); and 4 cases had the correct ICD codes, but not in the first or second diagnostic position. These findings highlight the rigidity of current criteria, which do not adequately account for variability in provider practices, particularly for pediatric populations.

This study evaluates only the PPV of the AFHSD surveillance case definition and does not include other metrics such as negative predictive value (NPV), sensitivity, specificity, or likelihood ratios. While these measures are important for a comprehensive understanding of case definition accuracy, calculating NPV was not feasible due to time and resource constraints. Conducting a sufficiently powered analysis would have required the chart review of over 2,000 cases to achieve approximately 80% power, which was beyond the scope of this study.

The findings from this study emphasize the need for continued refinement of surveillance case definitions for unique populations such as children. Potential solutions may include refining surveillance methods and modifying case definitions to incorporate greater flexibility for encounter timing and better account for variability in coding practices, as well as integrating data from specialized oncology centers and improving coordination of outpatient data. Future studies should explore these mechanisms to improve the accuracy and utility of surveillance case definitions in diverse populations and settings.

## Figures and Tables

**Table:** Chart-Confirmed True Positives, False Positives, and Unknown Cases Among All Cases Identified by AFHSD Malignant Brain Tumor Case Definition, Air Force Pediatric Dependent Population, Ages 0-19 Years, January 1, 2010–December 31, 2020

	True Positives		False Positives		Case Classification		
					Confirmed	Unknown	Total Cases
	No.	%^a^	No.	%^a^	No.	No.	No.
Total Cases	89	64.5^b^	49	35.5	138	41	179
Encounter type							
Inpatient	75	54.3	20	14.5	95	18	113
Direct care	43	31.2	11	8.0	54	11	65
Purchased care	32	23.2	9	6.5	41	7	48
Outpatient	14	10.1	29	21.0	43	23	66
Direct care	9	6.5	11	8.0	20	1	21
Purchased care	5	3.6	18	13.0	23	22	45

Abbreviations: AFHSD, Armed Forces Health Surveillance Division; No., number.

^a^Indicates row percentage of confirmed cases.

^b^Positive Predictive Value 64.5% (95% confidence interval, 55.9-72.5%)

## AUTHORS' AFFILIATION

Epidemiology Consult Service, U.S. Air Force School of Aerospace Medicine, Wright-Patterson Air Force Base, OH: Ms. Fryman-Wynkoop, Ms. Tacke

## DISCLAIMER

The views expressed are those of the authors and do not reflect the official guidance nor position of the United States Government, the Department of Defense, or U.S. Air Force. The presence of hyperlinks to external websites does not constitute endorsement by the U.S. Department of Defense of the linked websites, or the information, products, or services contained therein. The U.S. Department of Defense does not exercise any editorial, security, nor other control over the information that may be found at those locations.
